# Better Training for Matrons

**Published:** 1909-01-16

**Authors:** 


### Nursing Administration.

### BETTER TRAINING FOR MATRONS.

### II.—A MATRON'S DIPLOMA.

A curious dilemma is facing the nursing world at the present time. The demand for levelling up the standard of nursing, lengthening the course, and making it more thorough is being linked to a demand tending in exactly the opposite direction, for levelling it down to the requirements of a qualifying examination sufficiently easy not to discourage candidates of average ability. It is quite obvious that these two demands cannot be met by the same methods. The one militates against the other. The establishment of a standard of knowledge which shall be recognised by the State as sufficient for a nurse will instantly set stirring among probationers in general the conviction that it is waste of time to go beyond State requirements. In New York State the registration board requires at least two years' training. As a natural consequence the training schools which have been struggling to maintain a three years' course are heavily handicapped, and are in some notable instances beginning to give way. In England there is no doubt that the minimum course of training would be fixed at three years, and the hospitals now giving valuable fourth year extensions to their training would feel the pinch. The discouragement to higher training would be felt even more conspicuously in the subjects for examination. Qualifying examinations unless they exercise the mischievous effect of stifling a profession are necessarily very simple. It may be that however simple such a nursing examination might be, it would yet discourage good candidates from attempting to become nurses. But however this may be, it is certain that the standard of examination must be absolutely mediocre, and below what could be achieved by any woman of high intelligence who threw all her energies into her profession. As matters stand, therefore, there is grave danger that the higher courses of training may be rendered abortive, and the higher candidates discouraged if legislation is forced upon the nurse-training schools. The advocates for registration are alive to this difficulty, but they believe that it can be smoothed over by gradually forcing up the standard for the qualifying examination, and by providing for an order of "second-class nurses" to receive those who fall behind in the race. There is one thing certain, however, about a voluntary system of registration, and that is that no woman will submit to trouble and expense to brand herself as a second-class nurse. If it were possible to register nurses automatically on the expiry of their training it might well be arranged to issue two grades of certificates, but under a voluntary State system the proposal is ludicrously impracticable. The only course which can rescue nursing from deterioration should a low standard be established by law, would be the institution of a higher diploma for such nurses as desired to prepare themselves for administrative posts. The Nurses' Registration Bill has not yet become law; very probably it never will. But the matron's diploma need not depend on State legislation. All the progress which has marked the last fifty years in nursing matters has come from within the hospitals, not from without. Brilliant ideas, long-needed reforms have been brought forward experimentally in individual hospitals, have been proved, admired, imitated, at length become universal. Thus with a rate of progress which seemed tardy, but was in reality continuous, and therefore rapid, the voluntary institutions of this country have gone on their way unhampered by restrictions and free to inaugurate on a small scale alterations leading in the end to peaceful revolutions. We can already perceive signs that events are tending in the direction of the matrons' diploma. The ordinary course of study imposed on probationers in general is entirely inadequate to engross the faculties of able women, such as ought to be using their time to the best advantage in preparation for responsible duties in the future. During the first two years it is perhaps as well that there should be no differentiation. It is not every ambitious probationer with a good memory who has the qualities of a capable administrator. But at the end of two years the matron is able to form a very fair opinion as to whether her young nurses should be encouraged to advance to higher studies. Then for the next two years, when routine work has been thoroughly mastered, preparation should commence for the higher certificate. The curriculum ought not however to be too full for the nurse to undertake side by side with her ordinary ward work, or in the filling of such administrative posts as would widen her grasp of the special subjects. It is essential for the reality of the matrons' certificate that it should be awarded after actual work in the departments of the hospital and not separate the future matron from hospital experience by demanding a special collegiate course. The collegiate course has already been tried in America, where they are far more advanced in the theory and practice of matrons' work than this country. It has not, however, been a conspicuous success so far, and its value to the future matron's career may be estimated by the small number of those who enter for it. The break in continuity effected by entering for a collegiate course after training would probably act as an impediment to further progress in the nursing career. And to take the collegiate course first, and then sink to the level of the probationer is clearly out of the question. The only practical method is for the hospital to afford facilities for advanced work, within the walls. Undoubtedly if one of the leading training schools in this country would institute a course of training for matrons in harmony with the existing conditions of work, crowned with a special certificate of competence to undertake the duties of matron, it would meet with a very ready response.

